# Supplementary material for: A Global Screen for Assembly State Changes of the Mitotic Proteome by SEC-SWATH-MS
Source: Cell Syst. 2020 Feb 26;10(2):133–155.e6. doi: 10.1016/j.cels.2020.01.001 (PMC7042714; doi:10.1016/j.cels.2020.01.001)

P21283 | VATC1_HUMAN | ATP6V1C1 ATP6C ATP6D VATC
Monomer MW [kDa]: 43.942 Monomer expected elution fraction: 47

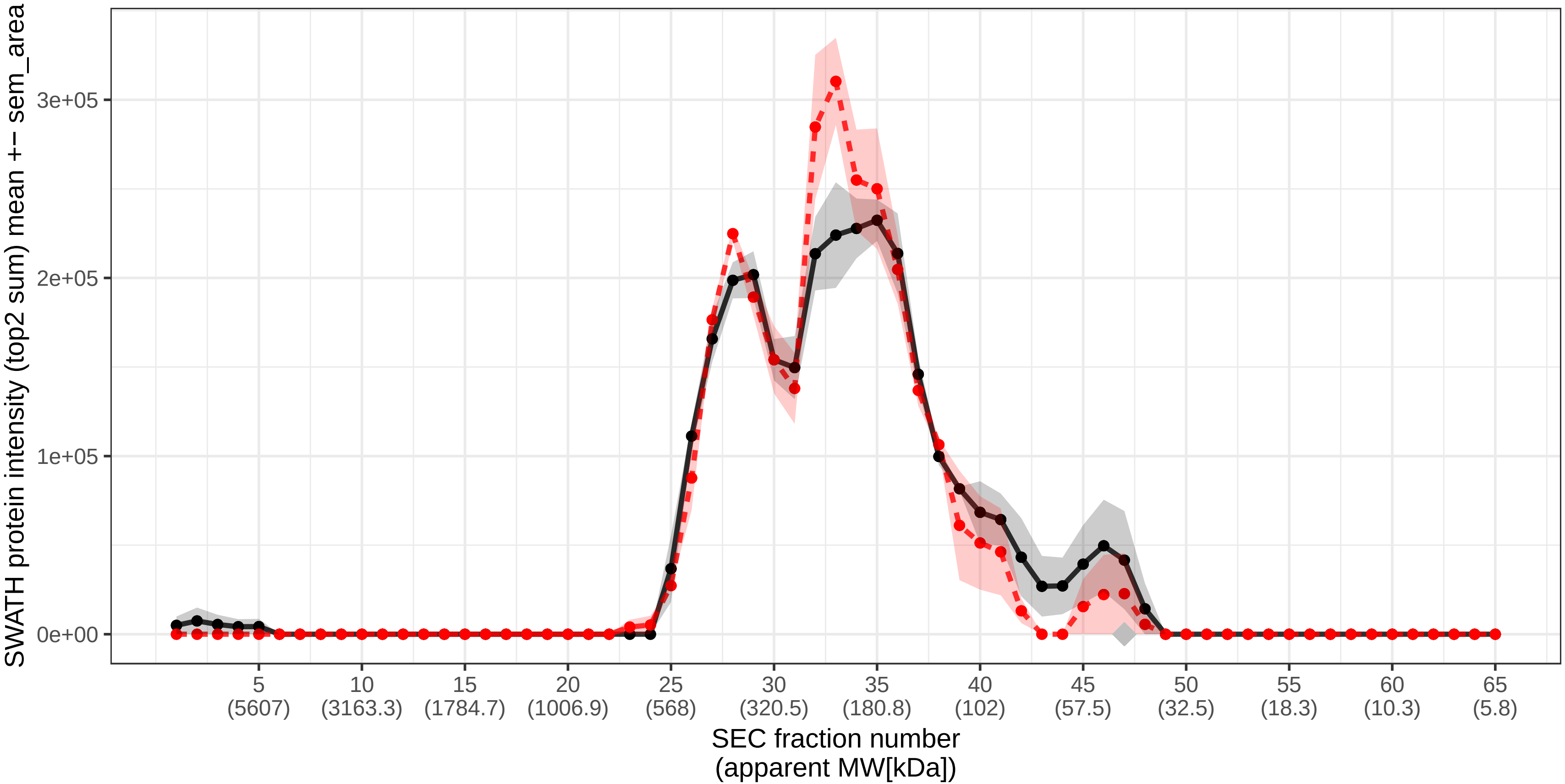

Supplement: Data S1. SEC-SWATH-MS Protein Chromatograms, Related to Figure 1 [file mmc6.zip › SECchrom_P21283_VATC1_HUMAN_ATP6V1C1_ATP6C_ATP6D_VATC.pdf]
